# Prostatic Adenocarcinoma in a Patient With a History of Cryptorchidism-Associated Hypogonadism: A Case Report

**DOI:** 10.7759/cureus.71158

**Published:** 2024-10-09

**Authors:** Haadia Safdar, Matin Sheriff, Javed Burki, Sher Shah Khan

**Affiliations:** 1 Urology, Medway NHS Foundation Trust, Gillingham, GBR

**Keywords:** bilateral congenital cryptorchidism, male hypogonadotropic hypogonadism, onco-urology, prostate cancer, testosterone (tt)

## Abstract

Prostate cancer is a prevalent malignancy often associated with advancing age and high androgen levels. Hypogonadism is characterized by low testosterone levels, and as prostate growth is androgen-dependent, this links elevated testosterone to increased prostate cancer risk. This rare presentation of a middle-aged man with the development of prostate cancer, following a history of congenital cryptorchidism and subsequent hypogonadism, challenges the conventional understanding of the role of testosterone in the development of prostate cancer. A gentleman in his late 60s, with a previous unilateral orchidectomy for suspected testicular cancer and 10 years of testosterone replacement therapy for hypogonadism, presented with an elevated prostate-specific antigen (PSA) level of 5.1 ng/mL. Digital rectal examination revealed a firm lobulated prostate, and MRI indicated a 15 mm PIRAD 4 (prostate imaging-reporting and data) lesion in the left peripheral zone. A trans-perineal biopsy confirmed a diagnosis of prostatic adenocarcinoma (Gleason score 3 + 3 = 6) with bilateral disease. To our knowledge, we are not aware of any previous literature reporting prostate cancer development in patients with cryptorchidism-associated hypogonadism. This report highlights the need for ongoing assessment of the long-term effects of testosterone replacement therapy in patients with a history of cryptorchidism and hypogonadism and the initiation or development of prostate cancer.

## Introduction

Prostate cancer ranks as the second most prevalent cancer among men worldwide, with increasing incidence and prevalence rates [1]. The disease can present in various forms, including asymptomatic cases discovered through screening, symptoms unrelated to the cancer, and patients who show apparent symptoms of the disease.

This type of cancer poses a significant health risk for men, with androgens, mainly testosterone and dihydrotestosterone (DHT), playing a vital role in its onset and progression. Androgens contribute their effects via the androgen receptor (AR), which stimulates the growth of prostate cells when activated. Elevated levels of circulating androgens have been associated with a heightened risk of developing prostate cancer [2,3]. These findings have led to the establishment of androgen deprivation therapy (ADT), a crucial treatment strategy for prostate cancer that focuses on lowering androgen levels or blocking their effects to slow tumor growth [4-6]. However, despite the recognized association between androgens and prostate cancer, debates continue regarding testosterone replacement therapy (TRT) in men with low testosterone levels. Currently, there is no convincing evidence in the medical literature that TRT causes the development of a new prostate cancer.

This situation underscores the need for further research to clarify the role of androgens in the risk of prostate cancer. Future investigations will be critical in refining treatment methods and enhancing outcomes for men diagnosed with prostate cancer.

## Case presentation

This case presents a 69-year-old gentleman with a history of being born with bilateral cryptorchidism and attempted bilateral inguinal orchidopexy at the age of 10. As per the patient, he continued to live with undescended testicles despite the attempted corrective surgery.

In his early 40s, he developed left-sided testicular cancer, for which he was seen at a different hospital and underwent laparotomy and removal of the left testis, followed by chemotherapy. In his late 50s, he was referred to the Urology Unit for the first time for progressively enlarging gynecomastia, on a background history of previous testicular cancer and cryptorchidism. He was thoroughly examined and found to have quite marked gynecomastia, with the right breast larger than the left and diffuse fibro-fatty tissue throughout both breasts. However, there was no evidence of axillary lymphadenopathy, hepatomegaly, or jaundice. He had no medical conditions or any drug history accounting for this presentation. His only medical history included hypercholesterolemia, for which he was on simvastatin, and asthma being managed with regular inhalers.

Given these findings, hormonal assays of follicle-stimulating hormone (FSH), luteinizing hormone (LH), prolactin, and testosterone were performed. The serum levels of testosterone were found to be less than the normal reference range, and the levels of LH and FSH were elevated, which led to establishing the diagnosis of hypogonadism in this patient. Table 1 presents the results of the hormonal evaluation performed.

**Table 1:** Hormonal profile at the time of diagnosis of hypogonadism FSH: follicle-stimulating hormone; LH: luteinizing hormone

Hormone	Result	Reference Range	Interpretation
Testosterone	6.9	10–30 Nmol/L	Low
FSH	51.9	0.9–15 U/L (male)	High
LH	35.2	1.3–12.9 U/L (male)	High
Prolactin	133	45–363 mU/L	Normal

Serum testicular tumor markers, such as AFP (alpha-fetoprotein), B-HCG (beta-human chorionic gonadotrophin), and LDH (lactate dehydrogenase) were also assessed, and all results were within normal limits. The prostate-specific antigen (PSA) level was also found to be within the normal range (0.9 ng/mL), making testicular cancer or prostate cancer very unlikely at that time.

Following the new diagnosis of hypogonadism, he was started on androgen replacement therapy in the form of topical testosterone gel and kept on close follow-up to monitor the serum testosterone levels. He was followed up for two years with an annual hormonal profile, which showed that he was maintaining adequate levels of serum testosterone and hence discharged to primary care for continuity of care.

**Figure 1:**
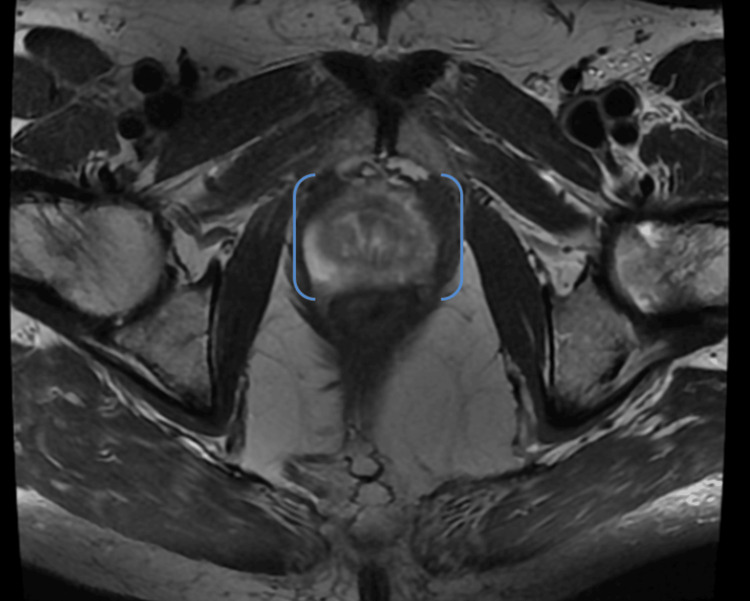
MRI pelvis showing prostate (bracketed in blue) with 15 mm PIRAD 4 focus noted in the left peripheral zone mid gland, with 82 mL gland volume and a PSA density of 0.062 PSA: prostate-specific antigen; MRI: magnetic resonance imaging; PIRAD: prostate imaging-reporting and data

In his late 60s, he was referred to our Specialist Urology Rapid Access Clinic for an elevated PSA of 5.1 ng/mL (doubled in one year from a PSA of 2.6 ng/mL in 2022) and a firm lobulated prostate upon digital rectal examination (DRE). MRI of the pelvis revealed a 15 mm PIRAD 4 focus in the left peripheral zone of the mid gland extending to the apex, with no enlarged pelvic lymph nodes (Figure 1). Prostate gland volume was assessed to be 82 mL, with a PSA density of 0.062. He then underwent trans-perineal prostate biopsy, which led to the diagnosis of prostatic adenocarcinoma with a Gleason score of 3 + 3 = 6, involving four out of the 27 submitted cores. Target biopsies were found to be positive, with bilateral disease.

Following discussions within the Multi-Disciplinary Team after a diagnosis of low-risk prostate cancer, the patient was offered all management options for prostate cancer. He has decided to be on active surveillance. He is being followed up with regular checkups and PSA tests as part of the active surveillance protocol.

## Discussion

Prostate cancer is the second most commonly diagnosed cancer in men, with an estimated 1.4 million diagnoses worldwide in 2020 [1]. The prostate is an androgen-dependent organ, and prostate cancer is an androgen-dependent disease [2]. Androgens are necessary for the development and normal functioning of the prostate, and consequently, there is a causal relationship between androgenic hormones and human prostatic carcinogenesis [3].

Cryptorchidism, or undescended testicle, is a condition where one or both testicles fail to descend from the abdomen into the scrotum. Men with cryptorchidism have a higher risk of developing testicular cancer. However, there is no documented link between cryptorchidism and prostate cancer. Surgical correction of undescended testis has the potential to influence hormonal dynamics affecting prostate cancer.

Men with androgen deficiency have reduced prostate volume and low serum PSA levels compared with their age peers [4]. Androgen deficiency partially protects against prostate disease, which has been previously used as the basis of the argument that cancer of the prostate is not seen in eunuchs (as they are incapable of developing functional prostates, benign prostatic hyperplasia (BPH), or prostate cancer) and that total androgen suppression by either surgical or chemical castration is a first-line treatment for advanced prostate cancer [5,6].

With advancing age in males, serum testosterone levels decline [7]. TRT is the mainstay of treatment for hypogonadism. Whereas androgen deprivation hinders the development and progression of prostate cancer and exogenous testosterone supplementation may possibly stimulate the growth of prostate cancer, it is unclear whether higher levels of serum testosterone are associated with a higher risk of prostate cancer [8]. A pooled analysis showed that men with very low concentrations of free testosterone have a below-average risk (OR: 0.77) of developing prostate cancer [9]. According to several studies and the recent European Association of Urology (EAU) guidelines, the consensus is that hypogonadal men receiving testosterone replacement do not have an increased risk of prostate cancer [10-12]. Notable among these is the TRAVERSE study, a randomized controlled trial [12] that mainly assessed the cardiovascular risks associated with TRT but also revealed that the incidence of prostate cancer in two cohorts of patients (receiving testosterone replacement vs. placebo over 22 months, followed up over a period of 33 months) was essentially the same.

As a safety precaution and for a holistic care approach, doctors prescribing testosterone supplementation should follow the patients with regular PSA concentration levels and DREs throughout the treatment period.

## Conclusions

Patients with a history of hypogonadism/TRT may develop prostate cancer. There is no supporting evidence that TRT causes prostate cancer initiation. Long-term follow-up and further research are crucial to elucidate the relationship between hypogonadism, androgen supplementation, and prostate cancer development. Furthermore, serum PSA testing and regular DRE checks are essential as a part of follow-up in patients receiving androgen replacement therapy.
